# Quantifying air quality co-benefits to industrial decarbonization: the local Air Emissions Tracking Atlas

**DOI:** 10.3389/fpubh.2024.1394678

**Published:** 2024-05-24

**Authors:** Amy B. Jordan, Daniel S. Rodriguez, Jeffrey A. Bennett, Kat Sale, Christopher Gilhooley

**Affiliations:** Carbon Solutions LLC, Saint Paul, MN, United States

**Keywords:** air quality, particulate matter, carbon capture, industrial emissions, decarbonization, co-benefits, public health

## Abstract

**Introduction:**

Many decarbonization technologies have the added co-benefit of reducing short-lived climate pollutants, such as particulate matter (PM), nitrogen oxides (NO_x_), and sulfur dioxide (SO_2_), creating a unique opportunity for identifying strategies that promote both climate change solutions and opportunities for air quality improvement. However, stakeholders and decision-makers may struggle to quantify how these co-benefits will impact public health for the communities most affected by industrial air pollution.

**Methods:**

To address this problem, the LOCal Air Emissions Tracking Atlas (*LOCAETA*) fills a data availability and analysis gap by providing estimated air quality benefits from industrial decarbonization options, such as carbon capture and storage (CCS). These co-benefits are calculated using an algorithm that connects disparate datasets that separately report greenhouse gas emissions and other pollutants at U.S. industrial facilities.

**Results:**

Version 1.0 of *LOCAETA* displays the estimated primary PM_2.5_ emission reduction co-benefits from additional pretreatment equipment for CCS on industrial and power facilities across the state of Louisiana, as well as the potential for VOC and NH_3_ generation. The emission reductions are presented in the tool alongside facility pollutant emissions information and relevant air quality, environmental, demographic, and public health datasets, such as air toxics cancer risk, satellite and *in situ* pollutant measurements, and population vulnerability metrics.

**Discussion:**

*LOCAETA* enables regulators, policymakers, environmental justice communities, and industrial and commercial users to compare and contrast quantifiable public health benefits due to air quality impacts from various climate change mitigation strategies using a free and publicly-available tool. Additional pollutant reductions can be calculated using the same methodology and will be available in future versions of the tool.

## 1 Introduction

Communities located in close proximity to areas with heavy industrial activity face multiple challenges, including air quality degradation, disproportionate climate change impacts, and environmental and economic injustices (1, 2). These crises have the potential to be simultaneously addressed using decarbonization technologies that not only reduce greenhouse gas (GHG) emissions but also mitigate short-lived climate pollutants such as particulate matter (PM), nitrogen oxides (NO_x_), and sulfur dioxide (SO_2_). Reducing these pollutants is expected to result in health “co-benefits.” These decarbonization co-benefits may deliver the most significant near-term benefits to people subject to poor air quality, which historically have not always been considered during policy analysis surrounding decarbonization and the clean energy transition (3).

Members of disadvantaged communities near industrial polluters frequently cite poor air quality as one of their top concerns (4), above many other economic, social, and environmental issues. In addition, communities may be hesitant to support certain decarbonization technologies, as these technologies can be seen as a means for industrial emitters to continue operating (5) or have other associated risks (6). This suggests that community buy-in to decarbonization projects might be improved through clear and transparent quantitative information about air quality co-benefits. Yet, such information is not readily available to many stakeholders or may originate from sources perceived as untrustworthy or have conflicts of interest.

There are several difficulties and data gaps that impede the calculation or estimation of air quality co-benefits from decarbonization for stakeholders and decision-makers seeking that information. In the U.S., the Environmental Protection Agency (EPA) databases that track GHG emissions–i.e., the Greenhouse Gas Reporting Program (GHGRP) – and other hazardous air pollutants–i.e., the National Emissions Inventory (NEI)–do not make use of identical facility information inputs such as tracking IDs, emitting unit names, or information regarding the industrial processes creating emissions. GHGs may be reported for facilities and their emitting units in different ways in the GHGRP database and the NEI, making it challenging to link GHG emission reductions to other co-pollutant reductions.

Decarbonization approaches such as carbon capture and storage (CCS), industrial electrification, and fuel-switching to low-carbon fuels have impacts on local air quality through various mechanisms. Even within one approach, such as CCS, the co-benefits can vary tremendously depending on the specific technology used (i.e., amine-based, cryogenic, membrane, sorbent, etc.) and the design specifications (7). This study focuses on amine-based carbon capture, as it is one of the most mature capture technology systems currently available (8). Because amine-based systems operate less efficiently in the presence of co-pollutants such as PM and NO_x_, pre-treatment of capture streams to remove these contaminants is essentially required (9).

On the other hand, amine-based systems can also result in an increase in certain pollutants, including ammonia (NH_3_), VOCs, and nitrosamines (10, 11). Once in the atmosphere, NH_3_ can lead to increased PM_2.5_ formation, potentially counteracting the co-benefits of PM removal (12). Similarly, increased VOC emissions may increase the production of secondary pollutants, such as ozone (13). However, technological options exist that can mitigate nearly all of the additional impacts to a facility’s emissions deriving from the use of amine-based carbon capture systems (14). Other types of carbon capture systems (e.g., cryogenic) may generate little to no new emissions (7).

Another factor impacting air quality is that certain decarbonization technologies, including CCS, cause an increase in energy use at an emitting facility, sometimes called the “energy penalty.” The emitting facility may see overall decreased pollution in return for additional pollution at the power source, which is seen in some life cycle assessments of CCS in some applications (15). In this study, we assume that the energy penalty is mitigated by the long-term migration to clean energy, a trend that is likely to occur in the U.S (16). This trend toward clean energy is predicted to cause air quality improvements throughout North America (17).

The LOCal Air Emissions Tracking Atlas (*LOCAETA*) is a free, publicly-available interactive analysis tool that quantifies and provides transparent information about possible emissions reductions of climate and local pollutants from decarbonization. The U.S. state of Louisiana was selected for demonstration of the methodology and results, as this state has the second-highest total industrial GHG emissions and the highest percent of its GHG emissions originating from industry compared to other U.S. states (18). This makes it a high-priority target for industrial decarbonization. Many of Louisiana’s heavily industrialized areas, including its infamous “Cancer Alley” (a region stretching from Baton Rouge to New Orleans, home to more than 200 industrial facilities), are subject to the highest percentiles of air toxics cancer risk and respiratory hazards in the U.S. (19). Many of these health burdens fall disproportionately on Black and impoverished communities (2, 20).

This research is of use to regulators tasked with selecting and optimizing climate change policies for their regions, to environmental justice communities seeking clear and transparent information about air quality improvements in their areas, and to industrial and commercial parties who are interested in pursuing decarbonization options with co-benefits. For example, one use case scenario could involve the use of *LOCAETA* in local (e.g., state, municipal) rulemaking efforts. By estimating emissions reductions from various facility decarbonization strategies, the public health impacts can be quantified, allowing for the inclusion of true costs communities experience from industrial emissions, and the associated economic benefits from decarbonization. Future versions of the publicly-available tool will directly quantify and display impacts to human health, which, along with clear and transparent discussions of uncertainty in the methodology, will provide users with additional valuable decision support that relies on co-benefits estimation.

Analyses aimed at quantifying health co-benefits from decarbonization are a growing area of interest and are represented by relatively few studies in the literature, most of which have been performed by researchers using advanced modeling software (21). The *LOCAETA* prototype and preliminary results presented here use a novel algorithmic approach to estimating the emissions reductions possible from one decarbonization option for industrial facilities (carbon capture and storage). As a free, publicly-available tool focused on industrial decarbonization and air quality at the community scale, *LOCAETA* will offer a unique combination of disparate datasets, analysis, and will also include new air dispersion and public health modeling in its future iterations. A review of other publicly available air quality visualization tools did not reveal any similar decision support tools like *LOCAETA*, which will ultimately (in its final form) show simultaneously the estimated emissions reductions for multiple decarbonization options, air quality impacts and co-benefits for specific industrial emitters, health risks and other demographic information, and satellite data, *in situ* sensor data, and modeling results. By considering air quality impacts alongside decarbonization strategies, we can maximize the benefit to public health and climate change mitigation, while empowering communities to actively participate in the process.

## 2 Methods

Because of the myriad of technological systems that may be employed in the pursuit of decarbonization, air quality co-benefits cannot be accurately forecasted across a wide scale of implementation and are typically estimated for individual cases where the system design and engineering is known. In the research presented in this study, which is necessarily screening-level, we assume a generic approach based on literature estimates of co-pollutant reductions from the flue gas pre-treatment required for amine-based capture. This allows facilities to be compared to one another across a broad geographic region, assuming the same decarbonization technology. This allows policymakers to optimize their efforts to combine decarbonization and air quality benefits, even though the realized mitigation will depend on the final system design and engineering specifications.

For the industrial facilities in Louisiana, amine-based CCS is one possible GHG mitigation choice that may be appropriate based on the area’s proximity to geologic sequestration resources and other major industrial activity along the U.S. Gulf Coast (22). CCS was selected for this study among other possible decarbonization approaches because there are preliminary estimates available of co-pollutant reductions necessary to efficiently operate typical amine-based systems (23), as well as estimates of undesirable emissions that may be generated. Because the decarbonization strategy must also be well-matched to the use case for any co-benefits estimates to be meaningful, the feasibility and affordability of CCS at individual facilities in Louisiana is also addressed in this research. This provides and links together important information that can help optimize facility selection for GHG and co-pollutant mitigation.

Original results are presented for estimated capturable CO_2_, alongside quantitative estimates of potential decreases in PM_2.5_ and increases in NH_3_ and VOCs for industrial and power facilities across Louisiana, from implementing carbon capture. These results are applicable only to CCS with amine-based technology, and to date, only for stationary combustion streams at the facilities. Stationary combustion sources generally are not a direct part of a dedicated process, such as cement calcination or iron ore reduction, and encompass a wide range of heat and steam production (although this heat or steam may be used in said process).

To estimate the co-benefits of amine-based CCS on industrial facilities across Louisiana, a crosswalk is needed between GHG emissions and other pollutants at a facility. For this analysis, only the impact of primary industrial PM_2.5_ emissions were selected because of the demonstrated effects of PM_2.5_ on public health, with major associated social costs (24, 25).

First, an analysis was performed to evaluate the feasibility and affordability of CCS across all Louisiana industrial facilities. This analysis breaks down facilities by processes (streams) of GHG emissions that are viable for capture. Next, those streams were matched to their corresponding emissions of PM_2.5_ from the EPA’s NEI database, using an algorithmic approach to overcome major dataset discrepancies and present an accurate one-to-one grouping of units and processes within each facility. Finally, the co-benefits from CCS on capturable streams were estimated using literature values of co-pollutant reductions required to operate a typical amine-based CCS system on an industrial facility. These methods are described in greater detail in the sections below.

### 2.1 CCS feasibility analysis

The CO_2_ National Capture Opportunities and Readiness Database (*CO_2_NCORD*) software uses techno-economic and life cycle modeling to evaluate the feasibility and cost of CCS (26). In this study, provides the total capturable MtCO_2_ annually from a facility’s total CO_2_, as well as the cost per ton captured. For industrial and power facilities in Louisiana, out of a total 141.5 MtCO_2_ of biogenic and non-biogenic CO_2_ emissions in 2021, an estimated 131.3 MtCO_2_ is capturable with an average cost of $63.19/tCO_2_. The capture costs can vary widely across facilities in the techno-economic modeling for Louisiana facilities, ranging from $18.68 to $71.13/tCO_2_ based on comparative literature-based cost estimates of capturing CO_2_ from specific facility- and unit-types.

*CO_2_NCORD* starts by extracting unit- and process-level emissions from GHGRP via the EPA EnviroFacts API (27), from the Emissions and Generation Resource Integrated Database (eGRID) (28), as well as additional sources for information about biorefineries and ethanol. Emissions are aggregated into capture streams based on EPA GHGRP subpart and when available, fuel type. If a facility reports to subparts H, S, or Q (cement, lime, and iron and steel, respectively), then *CO_2_NCORD* assumes that the stationary combustion streams are not separable from process streams. For example, a kiln at a cement facility will have emissions generated from both fuel combustion within the kiln (subpart C) and the chemical breakdown of the cement inputs (subpart H). These streams, while reported separately, are combined in the kiln and must be captured as one stream.

Identifying capture streams in this way allows *CO_2_NCORD* to develop a screening-level estimate of capture quantities and costs. The costs projected by *CO_2_NCORD* are meant to be an estimate given the annual emissions reported. A front-end engineering and design study would be required to determine site-specific capture retrofit costs for a given facility.

### 2.2 Facility-matching across EPA databases

A major challenge to co-benefits analysis is the lack of consistency of facility information between EPA datasets that compile reported emissions. GHG emissions used in *CO_2_NCORD* are reported from the facility to the GHGRP and eGRID under unique ID numbers specific to that program (e-GGRT ID for GHGRP and ORIS for eGRID). Co-pollutant emissions other than GHGs are reported to the NEI, which uses a different unique facility identifier known as the Emissions Inventory System (EIS) ID. For power plant facilities, eGRID is the most detailed source of GHG information used in this study, despite overlapping with some facilities in GHGRP and NEI.

The algorithm developed for crosswalking emissions from industrial units in GHGRP or eGRID to NEI is described in detail below. The conceptual approach is as follows. First, facilities must be identified as the same entity, and then, ideally, the same units within a facility can be linked across the datasets. However, because unit identifiers are highly irregular between the datasets, information about the processes performed by the units is used instead. To identify the same facilities between the datasets, ID numbers are used as much as possible; next, the algorithm seeks similarities in words present in (a) facility names, (b) parent company names, and (c) facility addresses, and also uses locations (reported latitude/longitude) to generate a score that indicates the strength of the connection between the two facilities. A cutoff value in this score is used to determine if two facilities across the datasets are in fact the same one. As discussed below, a QA effort was performed to manually check the success of the algorithm and choice of cutoff value. To finish the crosswalk between units within a facility, EPA subparts and source classification codes were used to identify and compare (in bulk) the streams estimated for CO_2_ capture and corresponding reductions in co-pollutant emissions, as described in Section 2.3.

The EPA’s Facility Registry Service (FRS) is a centrally managed database intended to track information across multiple datasets such as these and others; however, it frequently falls short of providing enough information to uniquely match facilities. A facility’s FRS ID is typically reported in GHGRP, but neither FRS nor e-GGRT IDs are used in NEI. NEI uses only EIS ID and sometimes a secondary code known as the Toxics Release Inventory (TRI) ID. These issues of non-matching facility ID codes would be inconsequential if facility/site names were uniformly reported across databases, but in practice, small and large naming differences are present between databases. More complex facilities may have reporting idiosyncrasies that confound easy matching, such as reporting to the GHGRP under one facility banner and reporting to NEI as different component parts of the same larger facility complex. For example, the ExxonMobil Baton Rouge Refinery and Chemical Plant (Louisiana’s largest emitter of PM_2.5_) reports as one facility in GHGRP (e-GGRT ID 1007643), but is split into a chemical plant (EIS ID 7226611) and refinery (EIS ID 8467211) in NEI. Also, addresses and point locations (longitude and latitude) frequently differ between the databases. Finally, because the NEI is only released every 3 years, and GHGRP and eGRID annually, mismatches may occur if different years are compared due to changes in facility name or ownership between years. In the case of this study, the *CO_2_NCORD* results reflect 2021 GHGRP and eGRID data and 2020 NEI data, so a small number of facilities may have changed names or ownership.

For this project, the goal was to identify co-pollutant emissions in NEI for every facility in which GHGRP data were used to calculate CCS potential within *CO_2_NCORD*. However, not every facility that reports GHGs to the GHGRP is reported in the NEI, and not every facility in the NEI is required to report to the GHGRP. Therefore, co-benefits cannot always be estimated between GHG reductions and co-pollutant reductions.

In Louisiana in 2021, the *CO_2_NCORD* analysis contained 415 facilities for which CCS potential was estimated. Offshore facilities which were evaluated for CCS potential in *CO_2_NCORD* were removed from the analysis of air pollution co-benefits because of their distance from population centers, as well as a frequent non-match to facilities in NEI. The remaining 310 facilities still required matching to their corresponding NEI database entries.

First, EPA databases containing facility information were used to match e-GGRT IDs to FRS, EIS, and TRI IDs, whenever possible. For the facilities in *CO_2_NCORD* for Louisiana, this resulted in a match between GHGRP and NEI only 21% of the time, with very high confidence in the accuracy of the match. Next, an algorithm was developed to associate facilities between the two databases based on similarities in site name, parent company name, and street address. A threshold was established for this automated facility-matching whereby a match was considered “reliable,” although still not as high confidence as matching through ID numbers. This resulted in acceptable matches for an additional 57% of facilities (not including the facilities previously matched with high confidence). Finally, for facilities that were still unmatched, a combination of proximity (through latitude/longitude) and similarity of name was assessed, to generate the final and least reliably matched facilities (3% of matches made by the algorithm).

The remaining 19% of onshore Louisiana facilities in the *CO_2_NCORD* results were thus unmatched between GHGRP and NEI by the algorithm. Quality assurance (QA) was performed to check the performance of the matching algorithm. One primary goal of this effort was to determine whether a large proportion of the GHGRP facilities that could not be automatically found in NEI were simply not present in that database. During the QA effort, manual matches could only be made in very few specific additional cases (4%). Most of the manual assignments were required due to the difference in facility names or locations between datasets (e.g., eGRID, GHGRP, and NEI). The remaining 15% of facilities did not have a match made; it is assumed they are not in NEI or have such divergent information between NEI and GHGRP that an algorithmic approach is not appropriate. Even manual matching would require further information than is available. The QA analysis suggested that many of the remaining unmatched facilities were compressor stations or other sites associated with oil and gas complexes.

The rest of the QA was focused on whether the algorithm described above resulted in probable false matches. The manual QA assessment of the algorithm determined that it had produced no false positives.

### 2.3 Matching CCS streams to emissions from units and processes

Because *CO_2_NCORD* calculates CCS potential using process information, it was necessary to develop an approach to align not just facilities but capture streams within facilities between GHGRP and NEI. For this initial study, we focused on EPA’s GHGRP Subparts C (general stationary fuel combustion sources) and D (electricity generation). Together, these comprise around 78% of the capturable CO_2_ estimated in *CO_2_NCORD* for Louisiana. At each facility, all streams evaluated in *CO_2_NCORD* that fall under Subparts C and D were combined, with the total MtCO_2_ capturable for the facility summed across the streams, and the weighted average cost per tCO_2_ captured for the facility was reported.

NEI does not include the same subpart or process information as GHGRP. However, it includes a set of process-related codes called the source classification codes (SCC). The SCCs were used to connect units in NEI that were likely the same ones associated with Subparts C and D in GHGRP. These were determined by identifying SCCs where the sector included “Fuel Comb,” the tier 1 description included “Fuel Comb,” or the third level included “Fuel fired equipment.” This approach is uncertain; for example, SCC 3999999 (“miscellaneous”) likely includes some probable fuel combustion processes, but other NEI units with that code may not. These units were not included *unless* a facility would otherwise have no units in NEI corresponding to a matched facility in GHGRP (e.g., facilities with e-GGRT 1004596 and 1,002,736). Finally, if the SCC indicated stationary combustion but the unit type or description indicated a non-capturable stream, these were excluded as they were also excluded in *CO_2_NCORD*. This included unit types of *flares* and *incinerators*, and all units with descriptions that included both the words *thermal* and *oxidizer* (as thermal oxidizer was not a recognized unit type in NEI, as compared to GHGRP).

Emissions from all units estimated as stationary fuel combustion in NEI by this method of using SCC codes and unit types/descriptions were summed for each facility, and assumed to represent a one-to-one correspondence to the capturable CO_2_ streams estimated by *CO_2_NCORD*. Uncertainties introduced by this method are described in the Discussion section.

### 2.4 Co-benefits estimation

To estimate the co-pollutant reductions, we followed the method from a study estimating the co-benefits of retrofitting CCS at emitters in Colorado (29). This approach used literature values to estimate the reduction in other co-pollutants captured in a traditional amine capture CCS approach, as well as to estimate a potential for increases in VOCs and NH_3_ from the system.

The projected decreases were reported for filterable and condensable PM separately, and were calculated as averages from case studies at two refineries and two cement plants (23) (Table 1). Negative emission reduction percentages correspond to increased emissions of VOCs and NH_3_ in tons relative to tCO_2_ captured. The estimated increase in VOCs was based on 3 years of measurements at the Petra Nova carbon capture project (23) and the potential increase in NH_3_ was estimated for an amine-based system as described in van Horssen et al. (11). Note that all of these estimated percentages may be significantly altered by the use of different systems or technology. Additional pollutant emissions due to the energy penalty of CCS is not considered here (10).

**Table 1:** Estimated capturable CO_2_, total PM_2.5_ and reductions, and VOC/NH_3_ increases possible from CCS of stationary combustion sources at four Louisiana industrial facilities.

Pollutant	Decrease (%)	Source
PM_2.5_ (filterable)	97	Brown et al. (23), average of Martinez, Beaumont, Mojave, and Buda facilities
PM_2.5_ (condensable)	94	Brown et al. (23), average of Martinez, Beaumont, Mojave, and Buda facilities
VOCs	−0.00022	Brown et al. (23), based on Petra Nova facility observations
NH_3_	−0.021	Horssen et al. (11)

Because emissions reductions in the literature were reported separately for filterable and condensable PM, those quantities were tracked separately and applied to PM_2.5_. As NEI reports total primary PM_2.5_ and filterable PM_2.5_, condensable PM_2.5_ is calculated as the difference between total and filterable PM_2.5_.

## 3 Results

Estimated PM_2.5_ emission reductions from carbon capture, potential increases in VOCs and NH_3_, and other related air pollution and public health data were combined in an interactive tool called the *LOCAETA* Data Explorer. The following sections describe the quantitative co-benefits analysis results for several key facilities in Louisiana and demonstrate the interactive tool.

### 3.1 Co-benefits analysis

**Figure 1:**
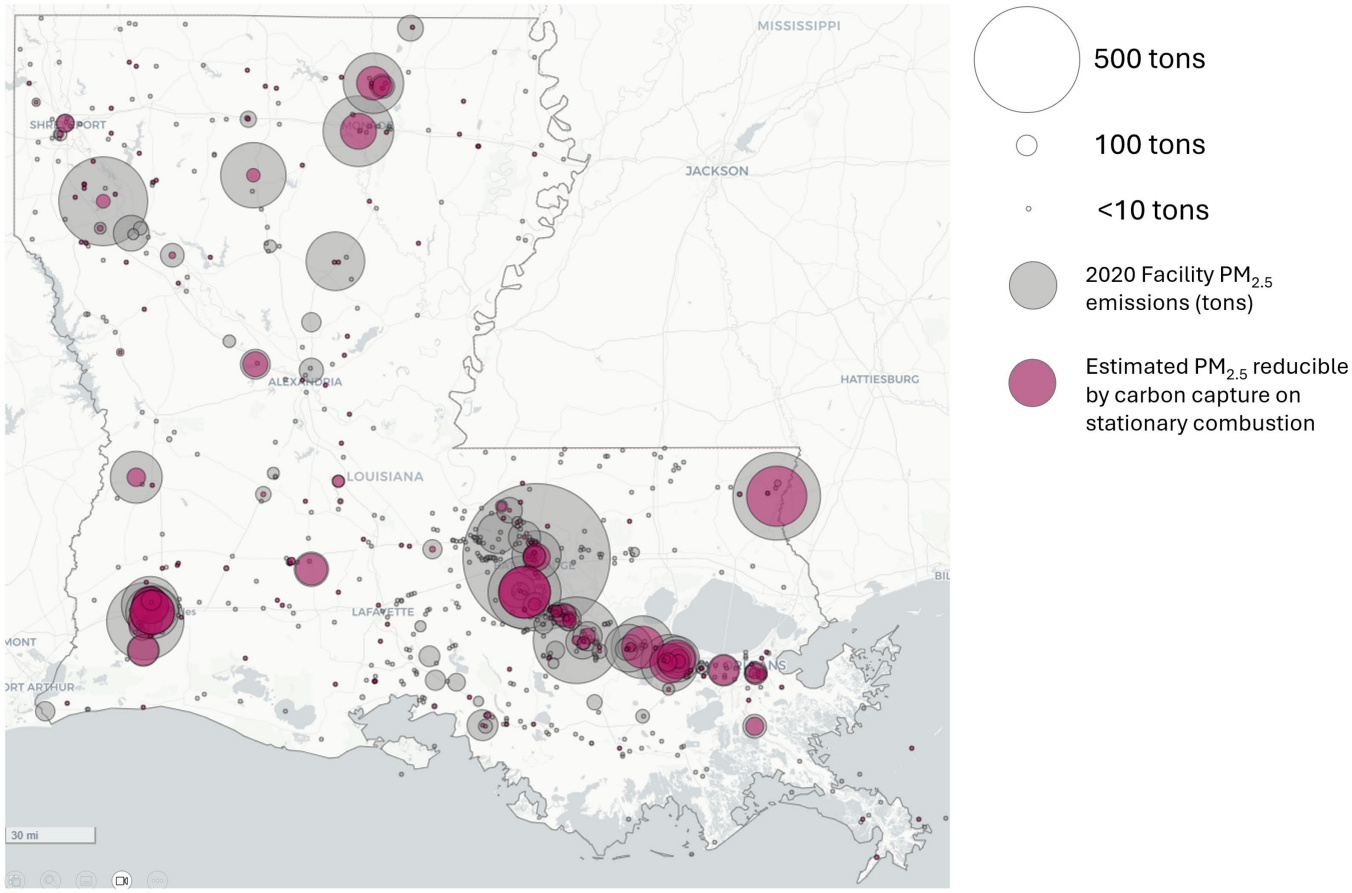
Facility PM_2.5_ emissions (gray circles, sized by tons of total facility PM_2.5_ emitted) and estimated reductions possible from carbon capture of stationary combustion (magenta circles, sized by tons of estimated reduced PM_2.5_).

The estimated potential PM_2.5_ reductions from carbon capture of stationary combustion sources at all industrial facilities in Louisiana are shown in Figure 1.

Several facilities in Louisiana were selected to present a snapshot of the co-benefits analysis results for different industrial sectors (refining, ammonia, chemicals, and pulp and paper). Louisiana’s largest PM_2.5_ emitter, the ExxonMobil Baton Rouge Refinery, is a large complex in East Baton Rouge that produces many types of petroleum-based fuels. The adjacent chemical plant is listed separately in NEI, but is combined in GHGRP, as described above.

The largest ammonia-producing facility in the world is the Donaldsonville Nitrogen Complex in southwestern Louisiana. This facility was Louisiana’s fourth largest PM_2.5_ emitter in 2020. The Dow Chemical Company’s Louisiana Operations facility, near Plaquemine, Louisiana, was also selected for comparison as an example from the chemicals sector. It was Louisiana’s sixth largest PM_2.5_ emitter in 2020.

The pulp and paper industry is also a prominent sector in Louisiana. Although many of its emissions are biogenic, as discarded wood products are frequently used as fuels in this industry, it offers many opportunities for CCS. The International Paper Co.’s Mansfield Mill in northwest Louisiana was the state’s second-largest PM_2.5_ emitter in 2020.

**Figure 2:**
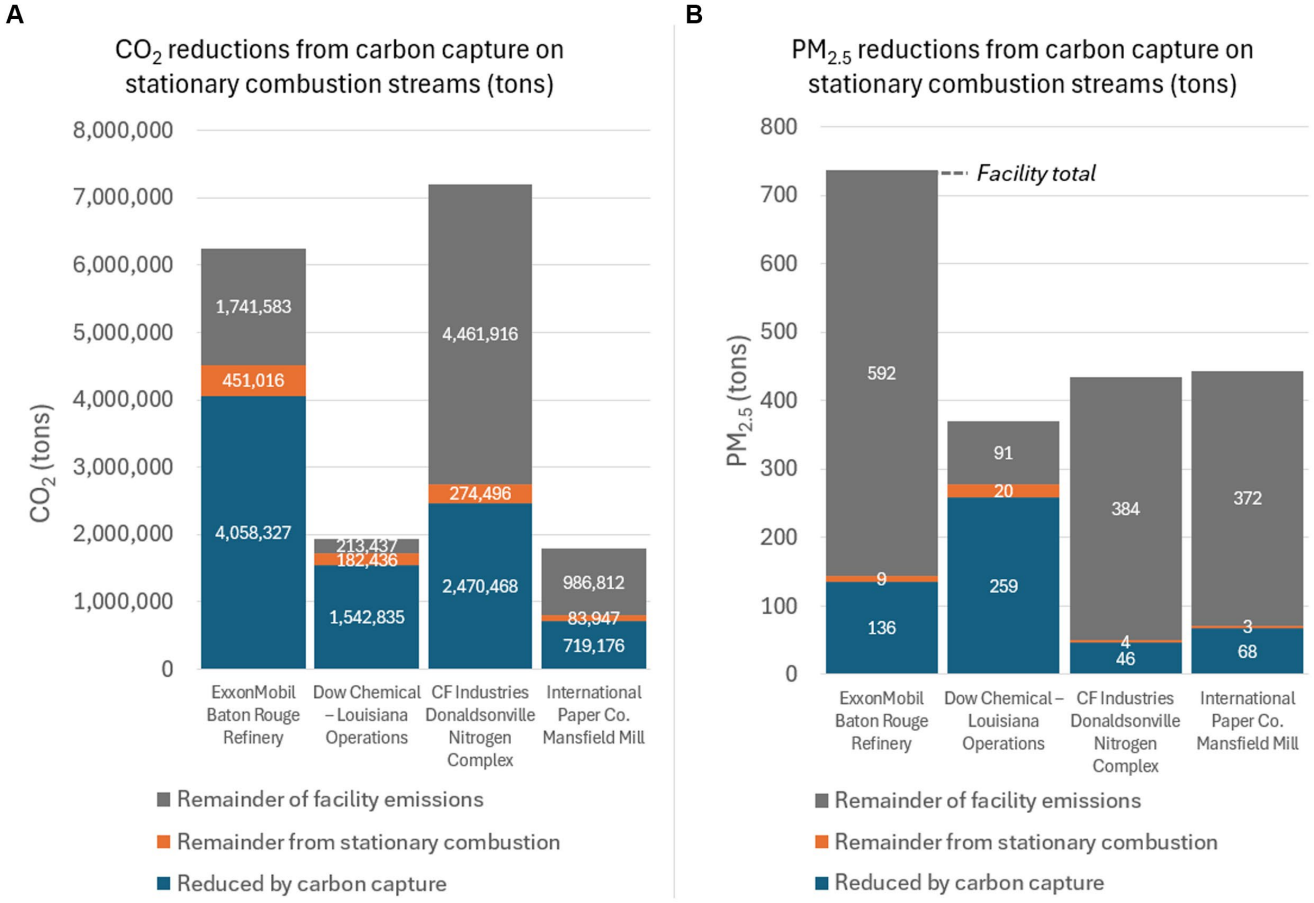
**(A)** CO_2_ and **(B)** PM_2.5_ emissions and reductions from amine-based carbon capture on stationary combustion streams at four Louisiana facilities. The remainder from stationary combustion represents the fraction of stationary combustion streams that is not considered capturable. The remainder of the facility totals are not stationary combustion, but may still be capturable.

Table 2 shows these four facilities, along with the estimated capturable CO_2_ and reductions in PM_2.5_. Estimates for the production of VOCs and NH_3_ from the CCS system are also provided. Figure 2 compares the facilities in Table 2.

**Table 2:** Estimated capturable CO_2_, total PM_2.5_ and reductions, and VOC/NH_3_ increases (in tons) possible from CCS of stationary combustion sources at four Louisiana industrial facilities.

Facility	tCO_2_ facility total^1^	tCO_2_ stationary combustion^2^	tCO_2_ reduced^3^	tPM_2.5_ facility total^4^	tPM_2.5_ stationary combustion^5^	tPM_2.5_ reduced^5^	tVOCs produced^5^	tNH_3_ produced^5^
ExxonMobil Baton Rouge Refinery	6,250,926	4,509,343	4,058,327	736	144	135	8.9	852
Dow Chemical–Louisiana Operations	1,938,708	1,725,271	1,542,835	365	274	254	3.4	324
CF Industries Donaldsonville Nitrogen Complex	7,206,880	2,744,964	2,470,468	432	48	44	5.4	519
International Paper Co. Mansfield Mill	1,789,935	803,123	719,176	444	72	68	0.9	89

Sources:

^1^U.S. EPA (40), 2021 Direct Emitters.

^2^U.S. EPA (27), https://data.epa.gov/efservice/C_SUBPART_LEVEL_INFORMATION/EXCEL

^3^CO_2_NCORD results (this study).

^4^U.S. EPA (37) 2020 NEI.

^5^LOCAETA results (this study).

These four facilities illustrate some differences between the impacts of co-benefits across different sectors. Statewide, 78% of capturable CO_2_ comes from stationary combustion streams. These selected facilities range from 34 to 79%, and were chosen to represent a range of outcomes instead of representativeness. The Dow Chemical Company’s facility offers the largest total and percent reduction of PM_2.5_ through capturable streams on its stationary combustion equipment, at 254 tons or 70% of the facility’s total PM_2.5_.

On the other hand, the Donaldsonville Nitrogen Complex has a much lower percent of total PM_2.5_ that would be reduced through carbon capture on the stationary combustion streams identified using SCCs (44 tons or just 10%). The wide difference comes in part from the difference in the facilities’ emissions (both GHG and other pollutants) that originate from stationary combustion, but also illustrate the need for further industry-specific classification of SCCs. In the case of the Donaldsonville facility, based on GHGRP classifications, the majority of emissions are process emissions from ammonia production, as opposed to from fuel combustion. Some of these emissions are from units known as urea granulators and ammonia reformers (30). These units are rejected from the stationary combustion streams based on SCCs, but the reformers may be a significant likely stream for CCS in the ammonia industry. Including the reformers at the Donaldsonville facility increases the estimated PM_2.5_ reduced from 44 to 213 tons, or 49% of the facility’s total PM_2.5_. Sector-specific analyses (e.g., ammonia, cement, pulp and paper) will help refine the remaining facility emission streams for CO_2_ capturability alongside co-pollutant reductions.

### 3.2 Interactive tool

The *LOCAETA* Data Explorer is built in JavaScript and is freely available to the public.^1^ It is a screening tool intended for users to better understand all sources of air pollutants that impact their communities, as well as to visualize the co-benefits offered by various decarbonization technologies. At present, the tool is set up to demonstrate only PM_2.5_ emissions data, sources, and estimated reduction potential from CCS in Louisiana. Other co-pollutants such as NO_x_ and SO_2_ will be added in future versions, along with nationwide scope and additional decarbonization approaches.

**Figure 3:**
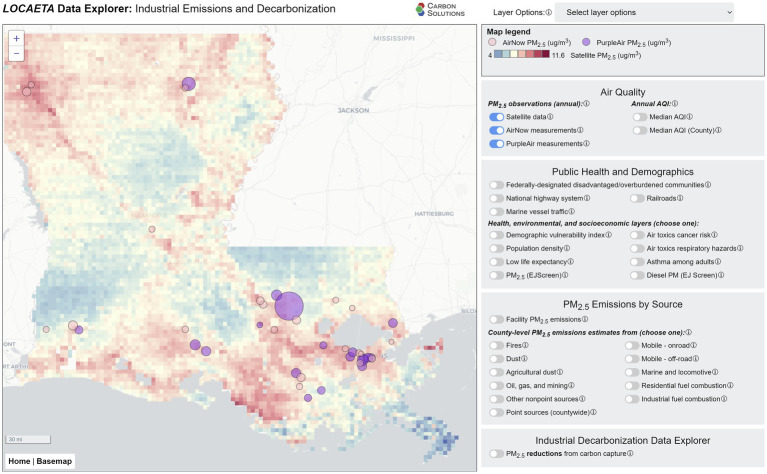
Screenshot from the *LOCAETA* Data Explorer showing satellite derived 2020 PM_2.5_ estimates for Louisiana, along with *in situ* monitors from AirNow (pink) and PurpleAir (purple) showing median 2020 emissions recorded at these monitors.

Figure 3 shows ground-level satellite-derived PM_2.5_ estimations in Louisiana in 2020 (31), along with 2020 median PM_2.5_ measured at *in situ* AirNow (32) and PurpleAir (33) monitors. PurpleAir monitors are constructed differently from AirNow sensors and typically record higher PM_2.5_ values (34). These layers allow users to see where PM_2.5_-measuring devices are located in their communities, as well as the estimated ground-level concentrations for the year across the state using the satellite-derived model results. The data shown represent yearly summary statistics, which may not convey the health risks for an individual in a community that receives stretches of poor air quality separated by stretches of good air quality throughout the year. Other layers, described below, show the estimated health and environmental risks associated with PM exposure.

**Figure 4:**
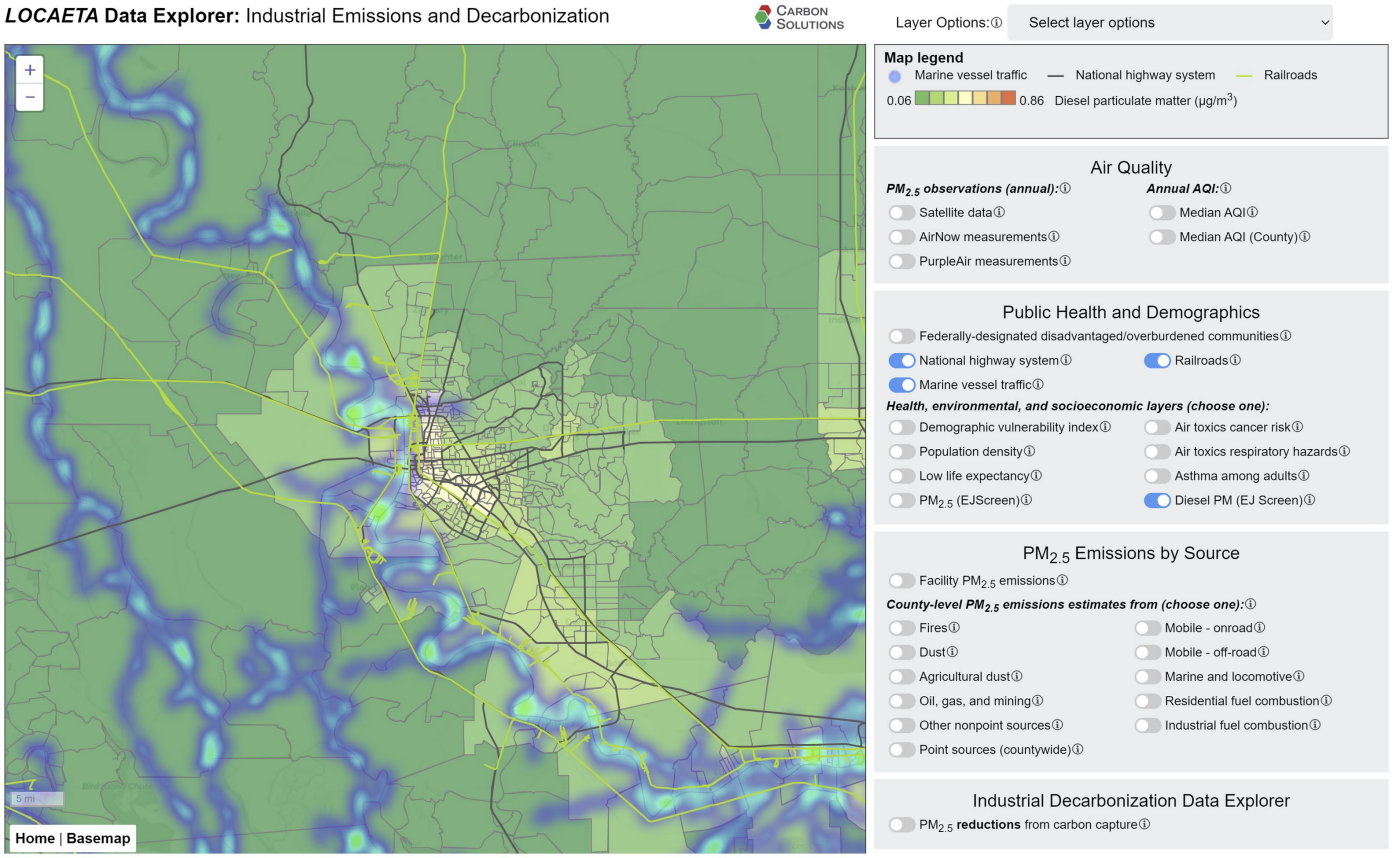
Screenshot from the *LOCAETA* Data Explorer, zoomed in to the Baton Rouge area, showing EJScreen diesel particulate matter, roads in the National Highway System (black lines), railroads (green lines), and marine vessel traffic (heat map).

There are many possible contributors to observed PM_2.5_ beyond primary PM_2.5_ emissions from industrial facilities. These include transportation (on road, offroad, marine, locomotive, etc.), fires, windblown dust, mining and petroleum extraction operations, residential fuel combustion, and other commercial operations not included in the industrial facilities reported under the NEI. It would also include generation of secondary PM_2.5_ from precursor pollutants (35). The *LOCAETA* Data Explorer allows the user to toggle through layers that show estimates of other distributed PM_2.5_ sources. Transportation-related layers are shown in Figure 4, zoomed in on the Baton Rouge area. Layers include diesel particulate matter estimated by EPA’s EJScreen (19), the location of highways and railroads, and estimated vessel traffic on the extensive waterways within the state. Most of these transportation methods use internal combustion engines, which are a significant contributor to total primary pollution in U.S. cities (36). In *LOCAETA*, the user can also select county-wide estimates of PM_2.5_ emissions from the types of distributed sources described above under the menu “PM_2.5_ Emissions by Source.”

**Figure 5:**
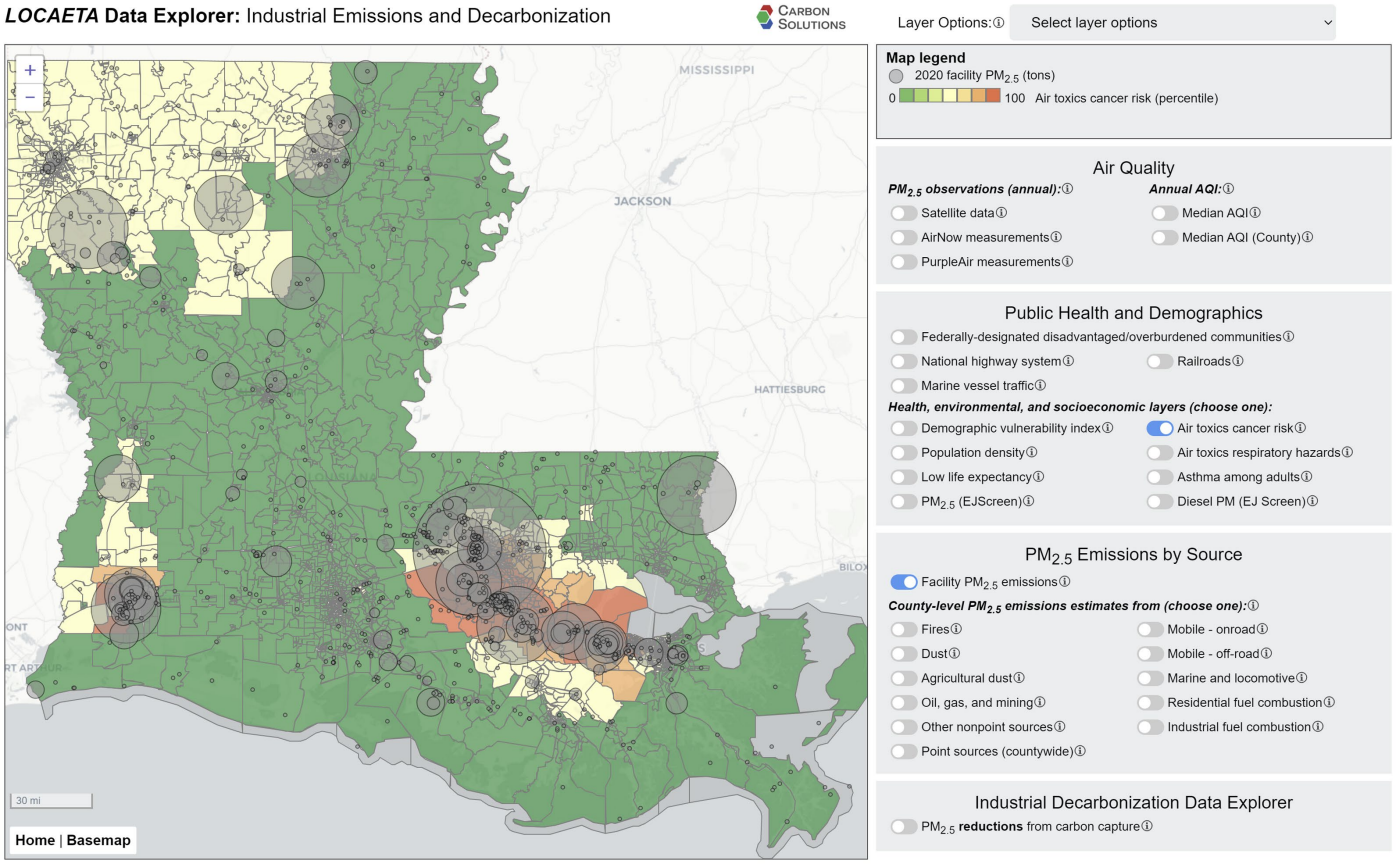
Screenshot from the *LOCAETA* Data Explorer showing EJScreen air toxics cancer risk percentiles and facility PM_2.5_ emissions.

Other layers in *LOCAETA* show the estimated or modeled health risks associated with all sources of toxic air pollutants. The air toxics cancer risk from EPA’s EJScreen is shown in Figure 5 (37). A region sometimes dubbed “Cancer Alley” is visible stretching from Baton Rouge to New Orleans in southeast Louisiana, where some of the highest nationwide percentiles of air toxics cancer risk is observed. This region is also home to much of Louisiana’s industrial PM_2.5_ emissions, as shown by the facility emissions layer. Other layers from EJScreen and the Climate and Economic Justice Screening Tool (CEJST) (38) that are presented in the *LOCAETA* Data Explorer include air toxics respiratory hazard index, asthma among adults, low life expectancy, population density, demographic vulnerability index, and federally designated disadvantaged communities.

## 4 Discussion

The results for the four facilities presented in detail here show that varying percentages of a facility’s total PM_2.5_ emissions are likely to be reduced, depending on the types of processes and capturable streams at the facility. The estimated potential increases in VOCs and NH_3_ vary in direct proportion to the estimated capturable CO_2_ from stationary combustion processes developed by *CO_2_NCORD*. The possible enhancement to VOC and NH_3_ emissions may moderate the overall realized co-benefits to air quality and public health from carbon capture. However, the extent of this moderation is highly dependent on temporal and spatial environmental factors. For example, the formation of PM_2.5_ from NH_3_ as a precursor is dependent on temperature and atmospheric composition and thus has a highly seasonal dependence (12). The exact effect enhanced VOC emissions would have on air quality also depends on the temporal and spatial context, as some seasons and locations are more sensitive to ozone formation (39).

The potential generation of negative air quality impacts associated with carbon capture should be considered whenever the positive co-benefits of CCS are touted, but technological improvements, including the adoption of zero- to low-solvent drift carbon capture technologies, may remove the necessity to consider these adverse impacts (14). Alternative forms of CCS will have different co-pollutant reduction requirements and may potentially have no pollutant increases (7). The energy penalty associated with CCS may be another factor impacting air quality in local communities (15), and this will be explored in future work, including when additional decarbonization options with associated energy penalties (e.g., industrial electrification) are added to the analysis and user interface in *LOCAETA.*

The quantitative co-benefits estimates presented here are necessarily a rough estimate and intended for screening purposes only. There are many uncertainties in the analysis and differences in technologies that would impact a facility’s typical air quality co-benefits experienced from installing a carbon capture system. Still, the ability to use a screening-level tool across a wide geographic region is of use to regulators at multiple levels (e.g., municipal, state, federal) when considering decarbonization decision-making and policy. More focused analyses at an industrial sector level would be of benefit in future work, because some of the simplifications and assumptions that are applied across all facilities could be refined for specific target industries (e.g., cement, ammonia, pulp and paper, refining, and other difficult-to-decarbonize sectors).

Uncertainties are introduced in this methodology by the facility-matching algorithm. It is unfortunate that this source of error exists, as simple changes in reporting requirements and methods for facilities to the U.S. EPA’s different programs would eliminate this issue (e.g., the use of agency-wide facility ID numbers). Some of the typical errors include false negatives (no match found between GHGRP and NEI for facilities that should have been found) and differences in the groupings of facilities where there are multiple facilities within a complex. False positives may also be introduced by the method, but a manual QA investigation found no false positives for these data for Louisiana in 2020/2021.

Further uncertainty is introduced in the step where units, processes, and capture streams within a facility are matched between *CO_2_NCORD* (which gives carbon capture potential and is based primarily on the GHGRP database) and NEI (which gives non-GHG pollutant emissions information). The goal is for the streams identified in *CO_2_NCORD* for carbon capture to correspond to the same set of units with emissions reported in NEI. For *CO_2_NCORD*, streams are identified by EPA GHGRP subpart (e.g., C, D) and fuel type. In NEI, a field called SCC is used. The 14,000 available SCCs include fairly detailed information about the type of processes, but there are inconsistencies in how facilities use SCCs and differences in the units that are ultimately grouped into streams between the databases. The categorization of SCCs into likely capturable stationary fuel combustion streams will be refined in future work.

The emissions reductions percentages used to quantify the PM_2.5_ co-benefits are taken from a small number of studies and may not reflect an accurate estimate for the complex streams and groupings of units with capturable CO_2_ within facilities. Nonetheless, the observation that a typical carbon capture system will require a relatively “clean” stream of CO_2_, with different tolerances for different contaminants such as PM_2.5_, SO_2_, and NO_x_, suggests that the approach of assuming pre-treatment and co-pollutant mitigation is sound. Actual amounts of co-benefits realized will depend on the technology and approach selected by the facility.

The estimates for potential increases in VOCs and NH_3_ are also uncertain. Different system types will result in varying levels of co-benefits and impacts. Future improvements to all capture types, including amine systems, will likely reduce potential air quality impacts directly caused by the capture process (14).

The *LOCAETA* Data Explorer allows users to compare these co-benefits quantitatively, with these assumptions and uncertainties in mind. It provides a reasonable screening-level comparison between facilities across industrial sectors. A typical user from a regulator or decision-maker’s perspective can quickly scan a region’s facilities and identify where CCS provides the greatest air quality co-benefits, with all else assumed equal about the systems employed. Future work will expand the tool to include other decarbonization options, from which patterns may emerge with respect to optimal approaches for certain situations. The user can also use the tool to concurrently visualize other sources and quantities of emissions that industrial decarbonization will *not* affect, such as those from transportation, dust, fires, etc. Finally, users can see public health and demographic data to see which groups are most strongly impacted by the intersecting problems of air quality health risks and other vulnerabilities that accrue in disadvantaged communities.

Future work on the *LOCAETA* Data Explorer includes improving the underlying analysis for quantitative estimates for decarbonization co-benefits from CCS and adding other options to the decarbonization menu, such as industrial electrification, energy efficiency optimization, facility decommission, and fuel-switching to other options such as green hydrogen. Each comes with unique challenges and potential externalities related to air quality, such as increased energy use. The tool will be expanded to U.S. nationwide coverage and include other pollutants besides PM_2.5_. Screening-level atmospheric modeling will be performed to allow more accurate estimates of the true air quality benefits expected from facility decarbonization. Public health impacts modeling will also be performed and added to the interactive platform. The method could readily be expanded to cover global scope, with the primary consideration of difficulty being the level of disconnect between greenhouse gas and other co-pollutant datasets, as is the case in the U.S.

Often, technological advancements, such as the ongoing energy transition, do not prioritize environmental justice or equity. Communities comprised of lower socioeconomic status and/or primarily ethnic and racial minorities are historically the last populations to experience the benefits of major societal change. *LOCAETA* will help enable decarbonization decisions that advance the U.S. toward its climate goals while simultaneously benefitting air quality in disadvantaged communities.

## Data availability statement

The datasets presented in this study can be found in online repositories. The names of the repository/repositories and accession number(s) can be found at: https://apps.carbonsolutionsllc.com/locaeta/Data.

## Author contributions

AJ: Formal analysis, Methodology, Project administration, Software, Visualization, Writing – original draft. DR: Conceptualization, Data curation, Validation, Writing – original draft. JB: Conceptualization, Formal analysis, Methodology, Writing – original draft. KS: Formal analysis, Visualization, Writing – review & editing. CG: Visualization, Writing – review & editing.

## Footnotes

^1^ http://apps.carbonsolutionsllc.com/locaeta
